# Building lexicon-based sentiment analysis model for low-resource languages

**DOI:** 10.1016/j.mex.2023.102460

**Published:** 2023-10-22

**Authors:** Idi Mohammed, Rajesh Prasad

**Affiliations:** Computer Science Department, African University of Science and Technology, Abuja, Nigeria

**Keywords:** Lexicon dictionary, Low-resource languages, Sentiment analysis, Fine-tuning, Hausa language, Building a lexicon-based sentiment analysis Model for low-resource language

## Abstract

Natural Language Processing (NLP) has transformed machine translation, sentiment analysis, information retrieval, and conversation systems. NLP applications rely on complete linguistic resources, which might be difficult for low-resource languages. NLP solutions for every language require a language-specific dataset. Dataset in a language is essential for NLP solution creation. Over 7000 languages are spoken worldwide. Only around 20 languages have text corpora for NLP applications. English has the most datasets, then Chinese and Spanish. Japanese has several Western European language datasets. For an accurate NLP system, most Asian and African languages lack training datasets. To address this challenge, we propose a methodology for building a lexicon-based sentiment analysis model for languages with limited resources. The Hausa language was used as training and evaluation language. The methodology combines lexicon creation; augmentation, annotation, and fine-tuning model, and has been tested on a corpus of Hausa tweets achieving an accuracy of 98 %. The results suggest that our proposed model is a promising tool for sentiment analysis in a variety of applications, such as social media monitoring, customer service, and market research. Our methodology can be used for any low-resource language. The outline of the work done in this paper can be shown as follows:

• We propose a methodology for building a lexicon-based sentiment analysis model for languages with limited resources, using the Hausa language as a case study.
• The methodology combines lexicon creation, augmentation, annotation, and fine-tuning model, and achieves an accuracy of 98 % on a corpus of Hausa tweets.
• The results suggest that the proposed model is a promising tool for sentiment analysis in a variety of applications for low-resource languages.

## Graphical abstract

**Figure:**
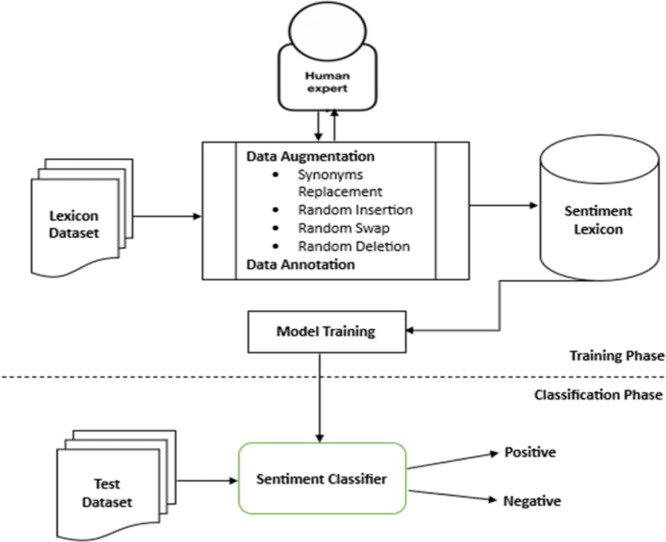

Specifications table

**Table:** 

Subject area:	Computer Science
More specific subject area:	Natural Language Processing
Name of your method:	Building a lexicon-based sentiment analysis Model for low-resource language.
Name and reference of original method:	https://www.tensorflow.org/tfmodels/nlp/fine_tune_bert[1]
Resource availability:	https://github.com/idimohammed/HausaBERT

**Method details**

## Introduction

Natural Language Processing (NLP) has revolutionized multiple aspects of human-computer interaction, including machine translation, sentiment analysis, information retrieval, and dialogue systems [2]. However, the effectiveness of NLP applications greatly depends on the availability of comprehensive linguistic resources, such as lexicon dictionaries and models, which can be a challenge for low-resource languages [3].To develop NLP solutions for any given language, the availability of data in that specific language is of utmost importance [4]. There exists a vast array of over 7000 languages that are spoken by individuals worldwide [5]. However, it is noteworthy that hardly 20 of these languages possess text corpora consisting of hundreds of millions of words. English has the most volume of data among all languages, with Chinese and Spanish following closely. In addition to Western European languages, Japanese is also among the languages that possess substantial databases. On the other hand, most Asian and African languages lack the necessary training data to construct accurate state-of-the-art NLP systems. From a technical standpoint, a language is classified as low-resource when it does not include extensive monolingual or parallel corpora, as well as manually created linguistic resources that are necessary for the development of statistical NLP applications [6]. To overcome this issue, we propose a methodology for building a lexicon-based sentiment analysis model specifically designed for low-resource languages. Lexicon-based methodologies are predicated upon the underlying concept that the semantic orientation of a given text is intricately linked to the polarity shown by the words and phrases included within it [7].The proposed model utilizes sentiment lexicons features to perform sentiment analysis in languages with limited resources.

## Literature review

This section reviews the scientific literature concerning the approaches related to building lexicon dictionaries for low-resource languages and sentiment analysis models for NLP applications that focus on low resource languages. The review starts with dataset articles followed by models’ articles.

### Lexicon dictionaries for low-resource languages

Chu et al. [8] proposed a method that employed statistical machine translation (SMT) models to generate translations for low-frequency words. By leveraging available parallel corpora, their approach significantly improved the coverage of the lexicon dictionary. Conneau et al. [9] introduced a methodology called “word translation without parallel data” that employed adversarial training to learn word embeddings in a shared space. This approach allowed for the generation of bilingual dictionaries without relying on parallel corpora, thereby benefiting low-resource languages. Sennrich et al. [10] introduced a method for extracting bilingual lexicons from parallel corpora, leveraging statistical machine translation techniques. Their approach demonstrated the potential of aligning parallel texts to generate lexicon entries. Caro and Rosado [11] emphasized the importance of involving domain experts and language professionals in lexicon development. Their study highlighted the benefits of expert knowledge in refining the lexicon entries and ensuring linguistic accuracy. In the paper “Unsupervised Transfer Learning for Multilingual Neural Machine Translation with Cross-Lingual Word Embeddings” by Mullov et al. [12], they proposed a method of unsupervised transfer learning for multilingual neural machine translation. Their method involves training a multilingual word embedding model on a large corpus of text in multiple languages. The multilingual word embedding model is then used to initialize a neural machine translation model for a new language, even if there is no labeled data available for that language. The results of the experiments in the paper showed that the proposed method was able to achieve significant improvements in translation performance for low-resource languages. The method was also able to generalize to new languages that were not included in the training corpus. Huang et al. [13] proposed an active learning approach that incorporated word embeddings to select informative words for human annotation. By iteratively refining the lexicon through crowd contributions, they successfully developed a comprehensive dictionary for a low-resource language.

### Sentiment analysis models for low-resource languages

Authors in [14] presents a sentiment analysis framework designed specifically for analyzing Indonesian tweets. The system can identify sentiments and opinions in each text or document. The model was constructed using a dataset consisting of four thousand tweets that were manually annotated. The categorization of tweets into eight distinct types is conducted due to the diverse range of information found within them. These groupings include positive (pos), negative (neg), and neutral (neu) sentiments, among others. Ultimately, the Long Short-Term Memory (LSTM) model achieved an accuracy of 73.2 % without a normalizer. In 2023, [15] develop SemEval-2023 in 2023. Task12: Sentiment Analysis for Low-Resource African Languages Using Twitter Datasets Trained on a collection of Twitter datasets for sentiment classification in 14 African languages. They employed various strategies, such as monolingual training, multilingual mixed training, and translation technology, and proposed a weighted voting system that combined the results of various strategies. The system attained Top-1 performance in two languages (Yoruba and Twi) and Top-2 performance in four languages (Nigerian Pidgin, Algerian Arabic, and Swahili, Multilingual) in the monolingual subtask. In the multilingual subtask, the system achieved a position in the top two on the competition. The authors of [14] report the use of SVM models in conjunction with ngram for sentiment analysis on Bangla news comments, achieving an accuracy of above 90 % with nonlinear SVM. In addition, the research conducted a translation of all emoticons into text and removed stop words. In 2017, [16] examines two approaches to Sentiment Analysis (SA) in the context of a low-resource language, namely Irish, with a particular emphasis on tweets pertaining to the 2016 General Election in Ireland. One approach involves the use of English sentiment analysis resources for the purpose of sentiment translation, which can be applied to both manually and automatically translated tweets. This technique has shown a level of accuracy reaching 70 %. The second approach is the development of a sentiment lexicon in the Irish language, known as Senti-Foclóir. This vocabulary is then used to construct the first Irish sentiment analysis system, referred to as SentiFocalTweet, achieving a commendable accuracy rate of 76 %. The research findings indicate that the process of translating from Irish to English has a limited impact on the preservation of sentiment. Additionally, the SentiFocalTweet system has been shown to be an effective foundational tool for analyzing sentiment in the Irish language. In 2021, [17] this work presents two significant contributions: BengSentiLex, a sentiment lexicon in the Bengali language, and BengSwearLex, a lexicon specifically focused on profanity in Bengali. The research presents a cross-lingual approach for developing BengSentiLex, which incorporates many components such as a machine translation system, a review corpus, two English sentiment lexicons, pointwise mutual information (PMI), and supervised machine learning classifiers. This study assesses the effectiveness of BengSentiLex in relation to translated English lexicons by using three distinct assessment datasets. The performance of BengSentiLex surpasses that of the translated lexicons, exhibiting an increase in accuracy ranging from 5 % to 50 %. BengSwearLex demonstrates a document-level coverage of around 85 % in an assessment dataset for the purpose of profanity detection. In 2018, [18] undertake a comprehensive examination of lexicon-based sentiment analysis in the Persian language. They offer two novel resources: PerLex, a meticulously annotated lexicon of sentiment terms, and PerView, a dataset consisting of about 16,000 items that have been assessed. The authors furthermore put forward a hybrid strategy that integrates machine learning and lexicon-based methodologies, using PerLex terms as training data for the machine learning algorithm. The PerView dataset was used for conducting experiments, and the findings indicate that PerLex exhibits superior performance compared to established lexicons such as CNRC, Adjectives, SentiStrength, PerSent, and LexiPers, in terms of both accuracy and execution time. The hybrid approach, which integrates the PerLex technique with machine learning, exhibits superior performance compared to both lexicon-based and machine learning approaches in isolation.

The literature review highlights various approaches and methodologies employed for building lexicon-based sentiment for low-resource languages for NLP applications. These approaches range from bilingual lexicon extraction and machine translation to cross-lingual word embeddings and active learning techniques. The involvement of linguistic experts and native speakers, as well as the utilization of resource bootstrapping methods, have proven crucial in addressing the challenges associated with limited linguistic resources. By building comprehensive lexicon-based sentiment analysis tools, we can empower NLP systems to effectively process and understand low-resource languages, fostering linguistic diversity and inclusivity in the realm of digital communication and interaction.

## Methodology

The methodology used in our study is based around the utilization of sentiment lexicons, which consist of compilations of words or phrases that are linked to corresponding emotion ratings. Sentiment scores are assigned to text documents based on the sentiment scores of the words and phrases they contain. This section describes the methodology used in building lexicon dataset and how the dataset can be used to fine-tune a BERT model for sentiment analysis.

### Lexicon compilation, augmentation, and annotation

**Fig. 1:**
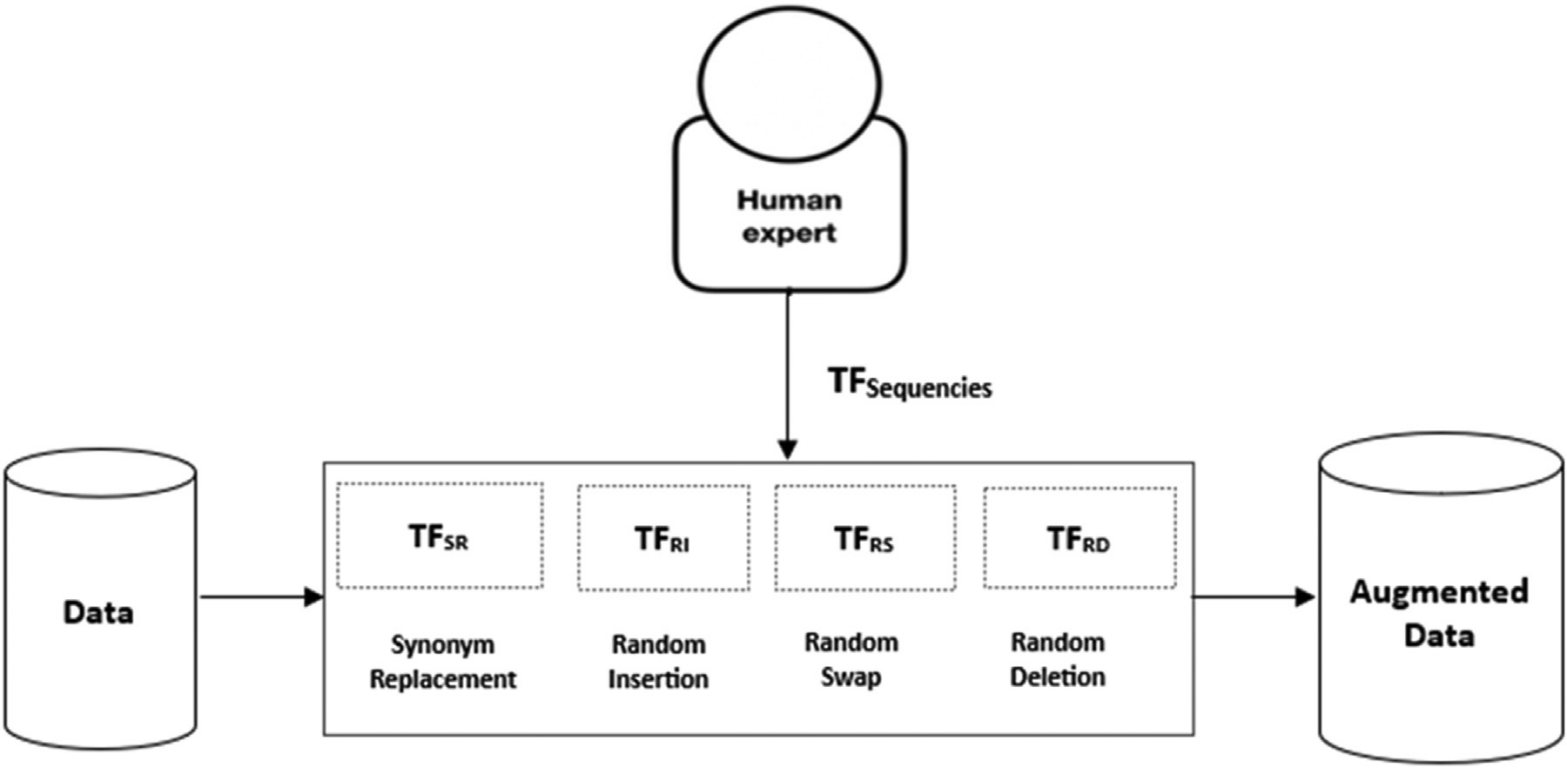
Data augmentation process.

This research develops a lexicon-based sentiment analysis dataset for the low-resource language (taken Hausa language as example) using data augmentation approach to be used as train and test data for the method implementation. Data was acquired from Hausa dictionary [19]. In the first instance, we identify list of words from the Hausa Language dictionary. In each word, we apply data augmentation procedures [20] which includes synonym replacement, random insertion, random swap, and random deletion to create sets of positives and negatives sentences or phrases which result in 13,392 lexicons. As shown in Fig. 1, data augmentations include the use of a deterministic series of transformation functions that have been fine-tuned by human experts. Five linguistics experts are employed for this task. Experts also perform data annotation. We validate the expert's agreement using the kappa statistic using formula in Eq. (1). The number of annotated sentences in each polarity group with their associated inter-annotation agreement are shown in Table 1.

(1) $$k=\frac{\mathrm{Pr}(a)-\mathrm{Pr}(e)}{1-\mathrm{Pr}(e)}$$

Where Pr(e) indicates chance agreement, while Pr(a) real observed agreement.

**Table 1:** Annotators agreement on positive and negative dataset.

Polarity	Records	k
Positive	6178	0.71
Negative	7214	0.69

The results of the annotators agreement are presented in Table 1. Initially 13,392 records containing 6696 each of positive and negative records were forwarded to the five linguistics experts to validate. All the annotators work separately. After completion, we tested if at least one choice was selected and the inter-agreement was more than 0.65, the sentence polarity will be accepted. After completion of this process, 518 positives words were amended and move to negative class.

Our augmentation process presented a dataset containing 6178 positives and equally 7214 negatives. Also, our datasets consist of bigram and above sentences of phrases. We also ignore neutrals words, as any text that is not categorized as positive or negative is automatically neutral. The augmented data will be used for training the model during implementation.

The annotated dataset available at https://huggingface.co/datasets/mangaphd/hausaBERTdatatrain

### Model fine-tuning

**Fig. 2:**
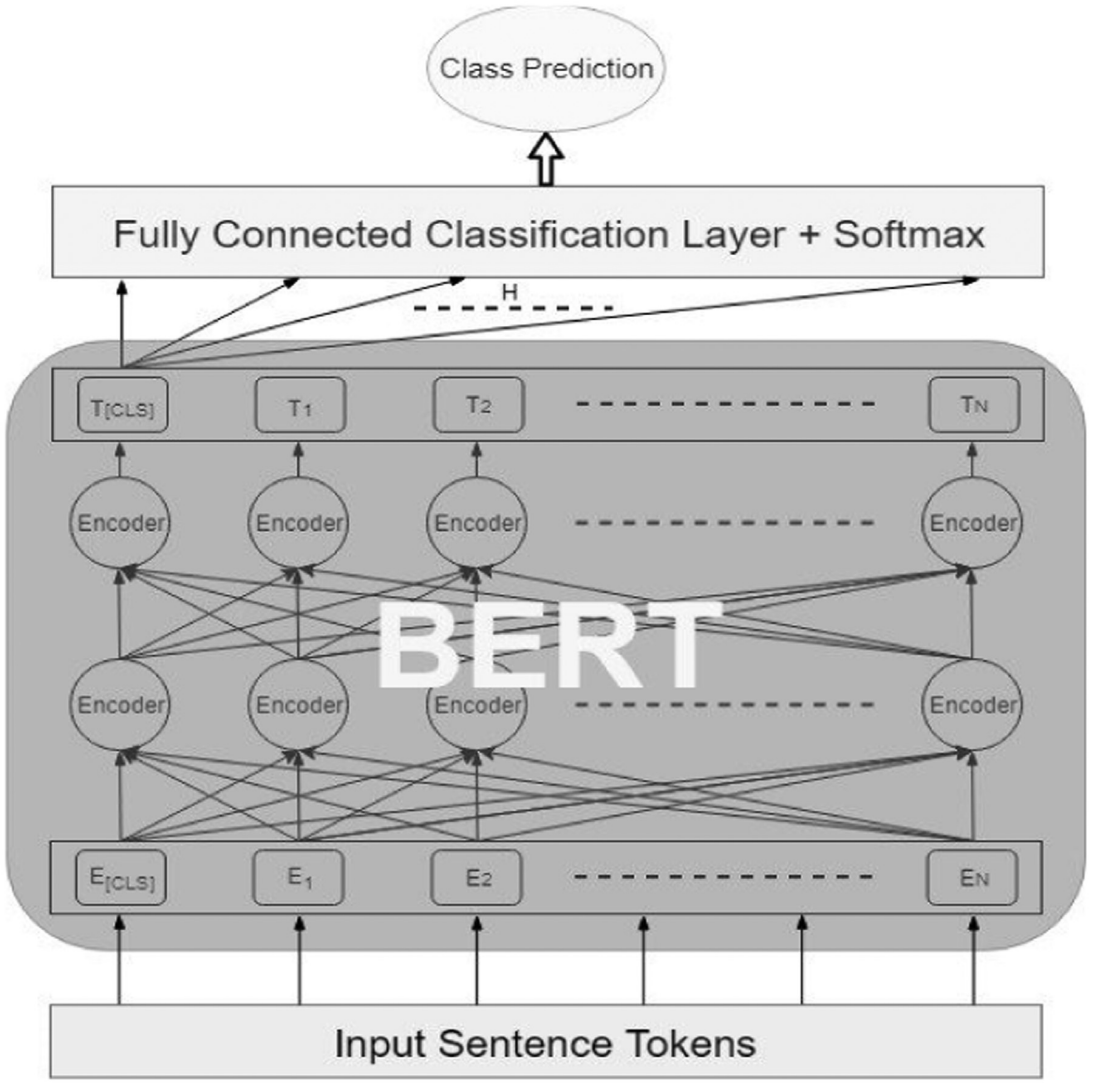
BERT fine tuning architecture [22].

We adopted a Transfer learning approach with Bidirectional Encoder Representations from Transformers (BERT) in [1]. Transfer learning is also referred to as pretrained model refinement [21]. It refers to training a model with a small dataset while leveraging the stored information from a model trained with a large dataset for a different task. Transfer learning is applied to a previously trained model by substituting its last layer with a randomly initialized new head for the new task. For a sentiment analysis fine-tuning model on a pre-trained BERT model, the head that classifies mask words will be removed and replaced with the sentiment analysis labels. As shown in Fig. 2, the classification layers are combined with softmax to provide class prediction module.

**Fig. 3:**
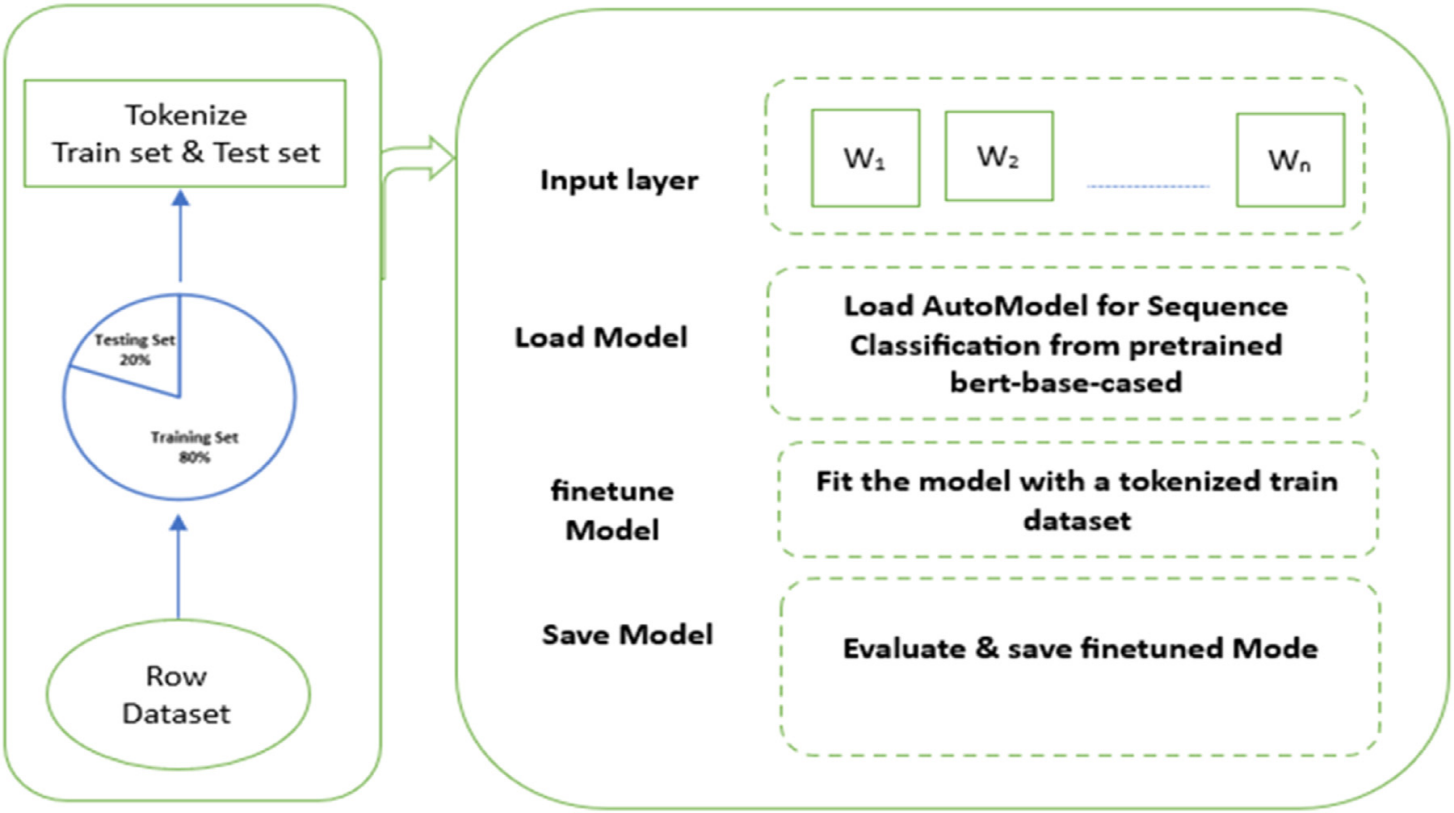
BERT finetune process.

The flow of the model fine tuning process is shown in Fig. 3. We provided a source code in Python for this method. Implementation is available at https://github.com/idimohammed/HausaBERTa

As shown in Fig. 3, we present three steps to accomplish the fine-tuning. The first step is to prepare the dataset. As explained in Section *Literature Review*, this software is trained with a dataset consisting of 9958 records with an equal number of positive and negatives labeled lexicons. The software loads the dataset as shown in Fig. 3, split it, and passed as input for pretrained process. 80 % of the datasets are used for training while the remaining 20 % are preserved as test datasets.

The second step is to load the BERT model that has been pre-trained. Download the pre-trained BERT model from Hugging Face without sequence classification head. As provided in Section *Comparison with existing models* of the source code, this code will load a pre-trained BERT model with 12 layers, 768 hidden units, and 12 attention heads. The model will have a classification head on top, which will be responsible for predicting the label for the input sequence. The model is trained with the tokenized dataset. We convert the tokenized dataset into a dictionary for Keras when fitting the model. batch_size = 4 indicates that four tokens are evaluated for each update to the weight and bias. epochs = 3 indicates that the model fitting procedure will iterate twice through the training dataset. Table 2 shows the training results obtained during fine tuning process.

**Table 2:** Training results.

Epoch	Accuracy	Loss
0	0.9168	0.2108
1	0.9385	0.1593
2	0.9549	0.1186

The Framework versions are 1) Transformers 4.33.2. 2) Tokenizers 0.13.3. 3) TensorFlow 2.13.0. 4) Datasets 2.14.5. 5) Tokenizers 0.13.3.

The third step is to evaluate the model performance on a held-out test set. This will give an idea of how well the model will generalize to unseen data. Our model scored above 90 % during the accuracy testing as shown in *Conclusion* of the finetune source code. As provided in the source code, the tokenizer.save_pretrained() and model.save_pretrained() methods are used to save the tokenizer and model, respectively, to the drive. This can be useful for sharing the tokenizer and model with others, so that they can use them for their own research or development. Our model achieves the following results on the evaluation set: 1) Train Loss: 0.1186. 2) Train Accuracy: 0.9549 and 3) Epoch: 2.

The final step is to publish the model for public use. We shared our model in huggingfacehubmodel hub using model.push_to_hub(“HausaBERTa”).

Our model is available at https://huggingface.co/mangaphd/HausaBERTa. Our complete package is share at https://github.com/idimohammed/HausaBERTa.

## Model description

**Fig. 4:**
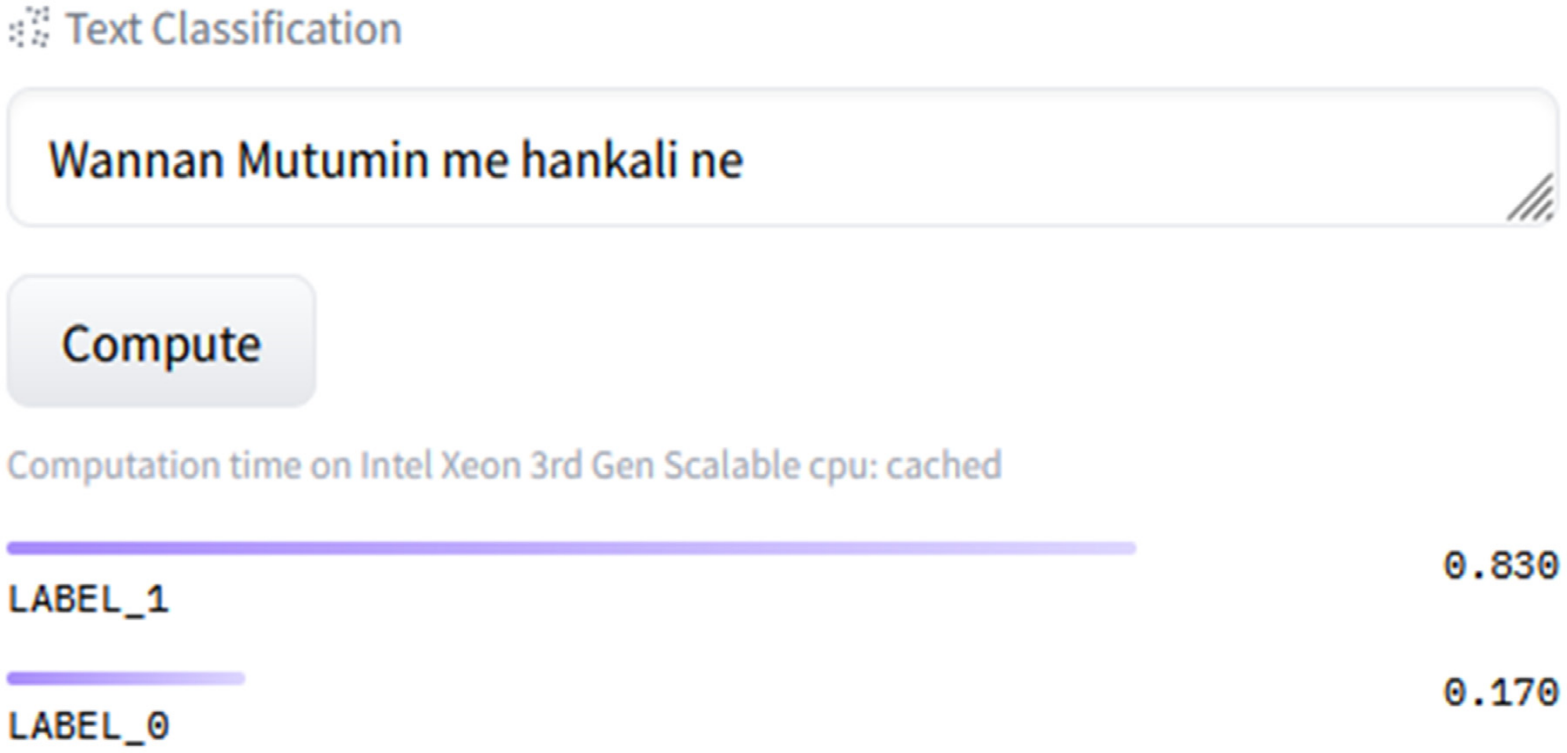
Huggingface Hosted inference API for HausaBERTa.

This section provides the description of the model and how to use it. Our model was implemented with Python using Google Colab. The model performance is limited to sentiment analysis on Hausa language text. The modelnamed HausaBERTa (Hausa Language Bidirectional Encoder Representations from Transformers) can be use directly on Huggingface hub. On the model card user can directly type in text and click on the compute button as show in Fig. 4. The API computes and displays LABEL_0 or LABEL_1 with associated confidence score. A confidence score is a value ranging from 0 to 1, which serves as an indication of the likelihood that the output generated by a machine learning model is accurate and capable of satisfying a user's query [23]. If the confidence score in LABEL_0 is above 0.6, this means the text is classified as negative. Likewise, if the confidence score in LABEL_1 is above 0.6 the result is considered positive. Neutral sentiment is detected if the confidence score of both LABEL_0 and LABEL_1 is below 0.6. We provided a python code for the sentiment interpretation in our model card.

Using the model directly by using the source codes requires environment setup which depends on user need. As show in Fig. 4, our model can be used directly from the huggingface transformers library.

**Fig. 5:**
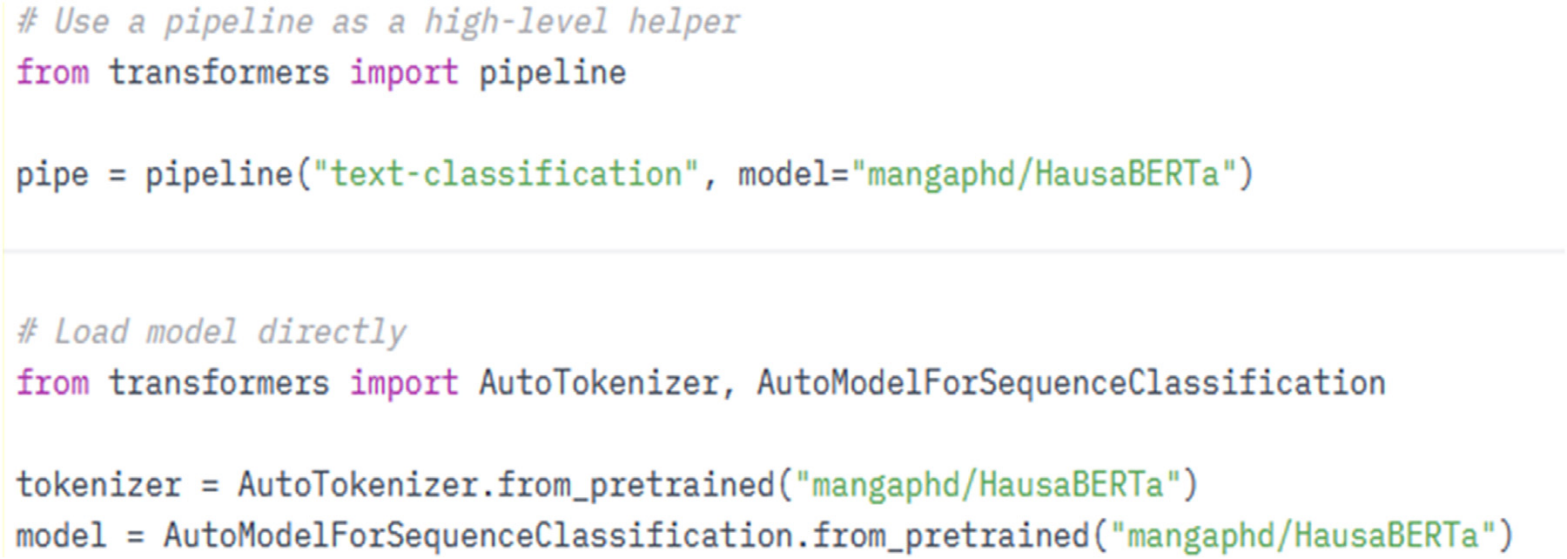
Model loader.

As shown in Fig. 5, the user can load the model from pipeline or load it directly. Similar output is generated by the code as that of Huggingface hub. Finally, this model can also be use using csv file as input. In our python source code (HausaBERTa_bulk.ipynb) we outline six steps to perform sentiment analysis on tweets save in csv file. The steps are:

i. Import the required Python packages: this loads and install the requires Install libraries for sentiment analysis. These include transformers, pandas, and colorama.
ii. Creates a pipeline object for text classification. This module loads HausaBERTa model from huggingface model hub. The loaded model does the sentiment analysis task.
iii. Prediction Interpreter: As mentioned in model card, the model output requires interpretation based on user requirement. This module converts the model output to either positive, negative, or neutral.
iv. Main: The main module reads csv file and passes each row to sentiment analysis model (HausaBERTa) through interpreter module until end of file. Its combine tweets with polarity using data frame.
v. Save: This module saves the data frame generated in the main module to csv file as output.

This section described the three methods this model can be accessed and use. These include using it directly from huggingface model hub, connecting via pipeline and uploading bulk text. Working directly requires the user to type in text as input. The pipeline method give flexibility to user to specify how to pass input and customize output interpreter confidence score. Working in bulk allows user to test bulk tweets or text and get output as file for further analysis.

## Comparison with existing models

We compare our software performance with TextBlob, VADER (Valence Aware Dictionary for Sentiment Reasoning), Flair, and BERT-base-multilingual-cased-finetuned-hausa. TextBlob, VADER, and Flair are libraries for NLP that can be utilized for sentiment analysis. TextBlob is trained on a variety of English corpora, including the Penn Treebank Corpus and the Brown Corpus [24].VADER is trained on a corpus of social media text, such as tweets and product reviews [25].Flair is trained on a variety of English corpora, including the Universal Dependencies Treebank and the Wikipedia corpus [26]. We also included in our comparison model that is trained on African languages which Hausa is among Davlan/naija-twitter-sentiment-afriberta-large (AfriBERTa). We provide a source code at https://github.com/idimohammed/HausaBERTa comparison with implementation in Python for our HausaBERTa with the mentioned NLP libraries and fine-tuned BERT models which perform similar task with our model. TextBlob, VADER, and Flair mostly regarded as general classifiers while the BERT model is limited to the African language. Table 3 shows the comparison of HausaBERTa with four other related models.

**Table 3:** HausaBERTa performance with related Models.

Model	Dataset			Accuracy (F Score)			
	Name	Size	Language	Reported by author	Test data set		
					1	2	3
HausaBERTa	hausaBERTdatatrain dataset	13,394	Hausa	98	89	52	87
TextBlob [30]	Variety of heuristics and rules	Large	English	unknown	0	29	6
VADER [25]	NLTK lexicon	Large	English	96	0	29	6
Flair [26]	CoNLL 2000, WNUT-17, WikiNER, etc	Large	English, German, French, etc.	unknown	24	30	29
AfriBERTa [27]	NaijaSenti	30,000	African multilingual	81	83	53	52

We use three datasets from public repository for the comparison. Hausa section of NaijaSenti dataset [27] was used as first testing dataset. NaijaSenti is a large-scale, manually annotated Twitter sentiment dataset for the four most spoken Nigerian languages: Nigerian-Pidgin,Igbo, Yoruba, and Hausa There are total of 3514 entries with 1755 positives and 1755 negatives tweets. The second testing dataset is ML Olympiad - Hausa Sentiment Analysis 2.0 [28]. There are a total of 256 entries with 94 positives, 75 neutrals, and 87 positives. The third dataset used for testing is CC100-Hausa Dataset by [29]. There are a total of 274 entries with 117 positive, 17 neutrals, and 140 negatives.

HausaBERTa, followed by AfriBERTa, Flair, VADER, and TextBlob, has the highest overall mean accuracy (F Score). The fact that HausaBERTa is the only model trained on a Hausa dataset suggests that it is more suitable for tasks involving Hausa text. AfriBERTa performance was excellent when compared with the other models in Test Dataset 2. Vader, TextBlob, and Flair performance in Hausa tweets are poor as shown in Table 3. Vader and TextBlob have get similar F scores of zero in test data 1, 29 on test data 2, and 6 each on test data 3. Although they are not train with Hausa text, but scores show that there is a significant fraction of code-mixed tweets on the test data set.

## Conclusion

In this paper, we present a methodology for building a lexicon-based sentiment analysis model for languages with limited resources. Our model leverages a lexicon-based dataset and bert-base-cased to fine-tune a low-resource language model for the Hausa language name as HausaBERTa. Our study shows the feasibility of building a precise sentiment analysis model for low-resource language, even when confronted with limited annotated dataset. We intend to extend our work in multiple directions in the future. First, we intend to investigate the use of alternative machine learning algorithms for sentiment analysis in low-resource languages. Second, we intend to improve the coverage of training dataset from Hausa Language other African languages like Zulu and Swahili.

## Ethics statements

*The dictionary used for testdata.csv file was collected from* [19] *with Author's consent.*

## CRediT authorship contribution statement

**Idi Mohammed:** Writing – original draft. **Rajesh Prasad:** Supervision, Validation, Writing – review & editing.

## Declaration of Competing Interest

The authors declare that they have no known competing financial interests or personal relationships that could have appeared to influence the work reported in this paper.

## Data Availability

Data is available at https://huggingface.co/datasets/mangaphd/hausaBERTdatatrain.
